# Provider perspectives on the provision of safe, equitable, trauma-informed care for intimate partner violence survivors during the COVID-19 pandemic: a qualitative study

**DOI:** 10.1186/s12905-021-01460-9

**Published:** 2021-08-27

**Authors:** Emma E. Williams, Kaetlyn R. Arant, Valia P. Leifer, Mardi Chadwick Balcom, Nomi C. Levy-Carrick, Annie Lewis-O’Connor, Jeffrey N. Katz

**Affiliations:** 1 The Orthopaedic and Arthritis Center for Outcomes Research, Brigham and Women’s Hospital, 75 Francis Street, Building for Transformative Medicine, Suite 5016, Boston, MA 02115 USA; 2 Community Health Intervention and Prevention Programs, Brigham and Women’s Hospital, Boston, MA USA; 3 Center for Community Health and Health Equity, Brigham and Women’s Hospital, Boston, MA USA; 4 Department of Psychiatry, Brigham and Women’s Hospital, Boston, MA USA; 5 Harvard Medical School, Boston, MA USA; 6 Division of Women’s Health, Brigham and Women’s Hospital, Boston, MA USA; 7 C.A.R.E. (Coordinated Approach To Resiliency and Empowerment) Clinic, Brigham and Women’s Hospital, Boston, MA USA; 8 Department of Orthopedic Surgery, Brigham and Women’s Hospital, Boston, MA USA; 9 Division of Rheumatology, Inflammation and Immunity, Brigham and Women’s Hospital, Boston, MA USA

**Keywords:** Intimate partner violence, Domestic violence, COVID-19 pandemic, Health systems, Qualitative, Care delivery, Telehealth, Trauma-informed care

## Abstract

**Background:**

Early research suggests the COVID-19 pandemic worsened intimate partner violence (IPV) in the US. In particular, stay-at-home orders and social distancing kept survivors in close proximity to their abusers and restricted access to resources and care. We aimed to understand and characterize the impact of the pandemic on delivery of IPV care in Boston.

**Methods:**

We conducted individual interviews with providers of IPV care and support in the Greater Boston area, including healthcare workers, social workers, lawyers, advocates, and housing specialists, who continued to work during the COVID-19 pandemic. Using thematic analysis, we identified themes describing the challenges and opportunites providers faced in caring for survivors during the pandemic.

**Results:**

Analysis of 18 interviews yielded four thematic domains, encompassing 18 themes and nine sub-themes. Thematic analysis revealed that the pandemic posed an increased threat to survivors of IPV by exacerbating external stressors and leading to heightened violence. On a system level, the pandemic led to widespread uncertainty, strained resources, amplified inequities, and loss of community. On an individual level, COVID-19 restrictions limited survivors’ abilities to access resources and to be safe, and amplified pre-existing inequities, such as limited technology access. Those who did not speak English or were immigrants experienced even more difficulty accessing resources due to language and/or cultural barriers. To address these challenges, providers utilized video and telephone interactions, and stressed the importance of creativity and cooperation across different sectors of care.

**Conclusions:**

While virtual care was essential in allowing providers to care for survivors, and also allowed for increased flexibility, it was not a panacea. Many survivors faced additional obstacles to care, such as language barriers, unequal access to technology, lack of childcare, and economic insecurity. Providers addressed these barriers by tailoring services and care modalities to an individual’s needs and circumstances. Going forward, some innovations of the pandemic period, such as virtual interactions and cooperation across care sectors, may be utilized in ways that attend to shifting survivor needs and access, thereby improving safe, equitable, and trauma-informed IPV care.

**Supplementary Information:**

The online version contains supplementary material available at 10.1186/s12905-021-01460-9.

## Background

While stay-at-home orders and social distancing helped mitigate COVID-19 transmission, these restrictions heightened safety risks for survivors of intimate partner violence and their children during the pandemic. (Intimate partner violence (IPV) is a term used interchangeably with domestic violence (DV). In an attempt to emphasize resiliency and empowerment, we refer to individuals who have experienced IPV as “survivors.” We acknowledge that no single term can fully describe a group and many survivors may not identify as such.) In the US, IPV affects one in four women and one in 10 men [1], and one in 15 children are exposed to IPV annually [2]. Recent research indicates that IPV severity worsened during the pandemic, as lockdowns forced survivors to remain at home with their abusers and reduced their abilities to escape or seek help [3–6]. COVID-19 mitigation measures also limited survivors’ access to support networks and resources, and introduced obstacles to screening for IPV in health care settings [7].

The increase in IPV during the pandemic is reflected in increases in IPV reports and calls. Analyzing crime data from 22 US states, Hsu and Henke report a five percent increase in IPV during the first two months of lockdown (March 13th to May 24th 2020) [3]. Similarly, police departments across the country have reported 10–27% increases in IPV reports in March 2020 relative to March 2019 [8]. These trends have also been observed worldwide. In France and Argentina, IPV increased 30% and 25%, and in Singapore, calls to IPV helplines increased 30% [6].

Many complex factors likely contributed to this increase in IPV, including financial strain and increased economic insecurity, increased stress, fewer interactions with mandated reporters, inability to screen for IPV during telehealth visits due to lack of guaranteed privacy, and greater difficulty filing restraining orders as a result of court closures [7–10]. The pandemic also introduced challenges for providers who care for or support IPV survivors. Telehealth visits limited providers’ abilities to assess survivors’ health and safety, and reduced the services they could offer. Social distancing exacerbated housing and shelter shortages, and lost wages due to the pandemic increased economic insecurity [10, 11].

We set out to understand the challeneges faced by providers involved in caring for IPV survivors during the COVID-19 pandemic, and the steps providers took to address these challenges. We conducted 18 interviews with providers involved in healthcare, housing, legal aid, and social work in the Greater Boston area, a US metropolis with more than 60 IPV shelters or programs [12] and a strong financial investment in combatting IPV [13, 14]. We analyzed their narratives qualitatively to understand the impact of the pandemic on care for IPV survivors and to generate hypotheses for future studies.

## Methods

### Participants and recruitment

We recruited individuals involved in caring for IPV survivors to participate in thirty-minute phone interviews. We used Google Search to identify organizations involved in care of and support for IPV survivors in the Boston area and then contacted individuals within those organizations. To be eligible, participants needed at least two years of experience working with IPV survivors prior to March 2020, with continued work during the pandemic. They also needed to work in the Greater Boston area and speak English as a primary language. Following an initial round of recruitment, we used key informants to suggest additional providers. The Mass General Brigham Human Subjects Committee approved the study protocol, and we obtained verbal consent from all participants.

### Interviews

Three authors (EEW, KRA, VPL) conducted the individual interviews with participants using a semi-structured interview guide (Table 1), which was informed by a literature review on COVID-19 and IPV. The interview guide included questions that covered challenges and barriers to care during COVID-19, adaptations and innovations developed to address these challenges and barriers, the implications of the pandemic on future policy and care, and lessons learned during the pandemic. The interview guide was created from a trauma-informed perspective, an approach to healthcare that considers the unique needs of survivors in all aspects of service provision [15]. We conducted and audio-recorded interviews from December 2020 through March 2021. Interviews were transcribed by an experienced transcription service. Interviewers met regularly throughout this period to minimize drift, and interviews were conducted until thematic saturation (the point at which no new information is learned) was reached. Each participant was offered a $40 check as remuneration.

**Table 1:** Topics included in the interviewer’s guide

Topic	Questions
Professional experience	1. Can you tell me a bit about your work and how long you’ve been doing it?
	2. Is there a specific organization you work for, or a specific community you serve?
Challenges and barriers	1. How has your work in identifying, caring for, and/or supporting IPV survivors changed during COVID-19?
	i. What challenges arose?
	ii. How are the changes in care you’ve observed during this pandemic similar or dissimilar to changes you may have observed during prior periods of stress, such as a natural disaster or economic downturn?
	2. How, if at all, did the pandemic exacerbate existing vulnerabilities within the network of individuals and organizations that identify, care for, and/or support IPV survivors?
	3. What barriers to accessing care or resources did COVID-19 create for individuals experiencing IPV?
Adaptations and innovations	1. How have the challenges you identified been addressed?
	i. What adaptations were made? What innovations emerged?
	ii. Were any pre-existing practices/tools repurposed? If so, how?
	iii. Do you believe any of these adaptations will remain long-term? If so, which ones and why? If not, why not?
	2. How has COVID-19 affected the screening process for IPV?
	i. What does virtual screening (i.e. telemedicine) look like?
	ii. How has in-person screening (i.e. ED visits) changed?
Broader implications	1. Do you think federal, state, or local government could have done more to predict or address the increase in IPV during COVID-19? Do you think private or non-profit organizations could have done more to predict or address the increase in IPV during COVID-19?
	2. How can government and private or non-profit organizations work together in the future, and what role should each play?
	3. How do you think COVID-19 has heightened inequalities in the access to and quality of IPV care and resources?
	4. How has COVID-19 impacted services outside of healthcare?
	5. How has COVID-19 impacted the coordination and cooperation between providers working in diverse sectors of IPV care (i.e. health care, social work, housing aid, legal services)? Do you believe any of these changes will endure?
Lessons learned	1. What are the lessons you’ve learned from COVID-19? How can they be applied going forward, in “normal” times or periods of stress?
	2. What is the advice you’d offer to individuals experiencing IPV? What are the most effective resources they can access during this time?
	3. What advice would you give to individuals who suspect IPV in a patient or social acquaintance?
	4. If you could go back to the beginning of the pandemic and make one change, what would it be? What resources would you ask for if you could have anything?

### Thematic analysis

We used thematic analysis, a common qualitative methodology, as described by Braun and Clarke [16]. Data analysis spanned three phases. First, three authors (EEW, KRA, VPL) developed a list of codes (keywords and phrases) describing the most basic elements of text that related to our study question: *how has COVID-19 impacted care for IPV survivors?* These three authors then coded all transcripts by assigning codes to all applicable segments of text using Dedoose, a qualitative analysis software (Dedoose.com, version 8.3.47b).

In the second phase of analysis, all authors reviewed a subset of transcripts and met to identify common themes and sub-themes across transcripts. Each theme encompassed multiple codes and captured overarching patterns in the data that related to our study question. Consensus was reached among all authors. Each theme and sub-theme was associated with at least one hypothesis, or explanatory statement describing its relationship to the study question, and supported by transcript quotations (Table 2).

**Table 2:** Themes, sub-themes (italicized), and selected supporting data identified through thematic analysis

Theme or sub-theme	Supporting text from transcripts
**I. Pandemic threat**	
Exacerbated external stressors	“I think it’s very similar [to an economic crisis] in that … folks that were basically on the cusp of survival now have fallen off that, … all of which we know increases stress within a relationship and then also increases vulnerability to intimate partner violence”
Restricted access to healthcare	“There’s no way to go to the doctor and speak with your PCP and tell them … ‘I’m in an abusive relatiionship’. No one is allowed to go to the hospital unless it’s a major issue”
*Fewer interactions with mandatory reporters*	*“We are very worried about that, about people not connecting with services, and we do know that DCF has received many fewer reports of child maltreatment during the pandemic because a lot of the mandated reporters are not seeing the kids”*
Fewer opportunities to leave abusive environment	“Our impression is that it’s worse, that people are not getting services and that they are trapped with their perpretrators in uncomfortable situations where people are stuck inside”
Fear of COVID-19 infection	“It felt like staff were concerned about their physical safety, so we’re able … provide services remotely”
	“We saw a very dramatic reduction in our volume of patients willing to come into the hospital…they were afraid of the virus”
**II. Community and system impacts**	
System-wide uncertainty and inconsistency	“We couldn’t keep up with who was supposed to do what in what court and how you applied…People just couldn’t navigate it…”
Strained systems and strapped resources	“[COVID is] just magnifying the issues that were already in place: housing insecurity, food insecurity, access to medical care, racism…”
Amplified inequities	“…racial inequality that has been exacerbated by COVID persists….My patients who live in certain communities I feel like have a harder time engaging in care”
*Language barriers to accessing resources*	*“…if they didn’t speak English as a first language, if they were unfamiliar with how to apply for it, if they didn’t know to ask for it and we didn’t know to offer it, they were inherently less likely to be able to apply for, be accepted to, and have access to emergency rent relief funding”*
Loss of community through isolation	“This is an isolating hard time for everyone but especially for somebody coming from a relationship where they’ve been isolated with… very little community support”
**III. Individual impacts**	
Heightened consequences of limited technology access	“…there is this expectation that like, ‘Oh, this is Zoom. Everyone knows how to do it. Everyone has that access to Wi-Fi at home,’ and that’s just not the case”
Complications of childcare	“I’ve talked with a lot of survivors who get stuck in this pickle if they are displaced from their house, they’re trying to maintain their job while also taking care of their children while being remote…”
Compounding trauma	“Survivors are survivors of intimate partner violence, but they’re also survivors of intergenerational trauma, community trauma…state-sanctioned violence on our communities”
*COVID as trauma*	*“When I think about survivors of domestic violence, and what they have already experienced when you add COVID and when you already add the trauma that they have already been living through, it just exacerbates depression, anxiety, and other mental health concerns as well”*
Deterioration of mental health	“People have much less access to one-to-one private interactions with their therapists or their psychiatrists…They’re so isolated…Many of them are just sort of fraying around the edges generally”
Strain on providers	“We’re in COVID. Everyone is getting sick. Everyone is overworked and underpaid. Everyone is trying to get used to this new way of lifestyle that we weren’t used to before”
**IV. Adaptations and innovations**	
Virtual interactions	“We have been using technology in a way that we didn't really use it before. We've been doing all our staff meetings using Zoom and we've all become very accustomed to speaking on Zoom”
*Flexibility of virtual encounters and remote work*	*“I would love that option to remain. I think that there is some nice—it’s nice to have a little bit of flexibility, especially when you’re working with trauma survivors who may not get to court on time or may be facing all sorts of other barriers that makes it hard for them to get to the court on time”*
*Difficulty building relationships virutally*	*“Really with DV survivors, you really do depend on building up a trusting relationship with people. It’s hard to do that without in-person—it’s just harder without in-person contact”*
*Privacy concerns*	*“On the other hand, if they’re sitting at home and they don’t know if their partner is overhearing or even if their kids are overhearing or even a random—or whoever, their parents or their group home housemates, I think they don’t have the same opportunity for privacy that they get in the doctor’s office”*
*Loss of networking*	“*It’s harder to network with somebody on Zoom. I remember I used to go to meetings or sit next to somebody and be like, ‘Oh, yeah. I actually have a client who speaks Russian and needs DV legal services. I’m so glad I sat down next to you,’ but that doesn’t happen”*
Importance of hybrid care	“I think having the choice is gonna be really important and being ble to have clear criteria around which those choce are made, because …different patients have different needs”
*Emphases on survival and emotional support*	*“Then after that, when people were home for a while, and it was just this monotony of unknown, we provided a lot of the emotional support, which we hadn’t anticipated to that degree”*
*A refocus on basic needs*	*“The other thing that we’re hearing is that they are also getting calls–when they do get calls, sometimes it’s for really basic stuff. It has nothing to do with the abuse.... No, it’s more, ‘We have no money. We both lost our jobs.’ They can’t even focus on the abuse right now. They’re just looking on to survive day-to-day”*
Willingness to Modify Practices	“We made it a point never to do telehealth before this…That’s quite different now”
Creativity	“I think creativity goes a long way in this work”
Cooperation and coordination	“…we’ve created something called the Boston Partnership. It’s a collaborative with other domestic violence, sexual assault, and support entities within the city of Boston, so some of the hospitals, clinics, legal advocacy services are involved…it’s a way for all of us to connect and adapt to the changing system of COVID…”

**Fig. 1:**
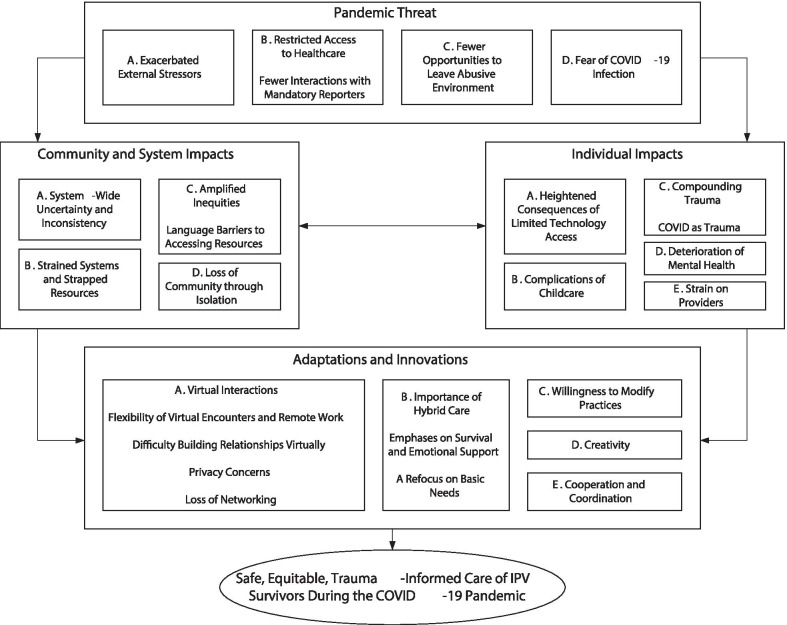
Thematic map

In the final stage of analysis, we constructed a thematic map, a visual representation of the relationships between all themes and sub-themes (Fig. 1). All authors were involved in developing themes and the thematic map.

## Results

### Participants

We sent recruitment letters to 59 providers in the Greater Boston area and called all individuals who did not opt out of the study. Of these individuals, 18 were reached and interested in participating. We conducted semi-structured one-on-one interviews with these 18 providers, who represented the following sectors of IPV care healthcare (n = 7), housing (n = 4), legal aid (n = 3), and social or community work (n = 4).

### Thematic analysis

Our thematic analysis identified 18 themes and 9 sub-themes, which we grouped into four thematic domains (Fig. 1). The four thematic domains included the external threat of the pandemic; the pandemic’s impacts on communities and systems; the pandemic’s impacts on individuals; and the adaptations and innovations that providers developed to address these impacts. Themes are described and presented with supporting quotations below. Following each quote, the sector of care in which the provider works is noted in parantheses. Select quotes are summarized in Table 2. A comprehensive thematic scheme is presented in Additional File 1: Appendix 1.

### Pandemic threat

The pandemic created many obstacles for IPV survivors, which led to an increased likelihood of abuse and/or reduced access to care.

A. Exacerbated External Stressors

The pandemic and its consequences (increased isolation and economic and housing insecurity) placed greater amounts of stress on individuals and relationships. These exceptional circumstances increased the likelihood of violence from abusers, *“I think it’s very similar [to an economic crisis] in that … folks that were basically on the cusp of survival now have fallen off that, … all of which we know increases stress within a relationship and then also increases vulnerability to intimate partner violence.” (healthcare).*

B. Restricted Access to Healthcare

COVID-19-related restrictions on capacity and transportation limited survivors’ access to health care providers and reduced their opportunities to seek help. *“There’s no way to go to the doctor and speak with your PCP and tell them … ‘I’m in an abusive relatiionship’. No one is allowed to go to the hospital unless it’s a major issue.” (housing).*

III. Fewer Opportunities to Leave Abusive Environment

Stay-at-home orders forced survivors to remain in close proximity to their abusers, and social distancing measures undertaken by institutions (e.g. hospitals, courts) reduced their abilities to access support systems and resources. Providers observed a “breaking point” several weeks into the pandemic when—in the absence of outlets survivors relied upon to cope with their situations, such as social support and work—survivors could cope no longer. At this point, many individuals sought help quickly, *“Our impression is that it’s worse, that people are not getting services and that they are trapped with their perpretrators in uncomfortable situations where people are stuck inside.” (healthcare).*

IV. Fear of COVID-19 Infection

The fear of contracting COVID-19 reduced survivors’ willingness to access care, and providers’ willingness to provide services. This promoted a quick transition to remote work and virtual encounters. Participants in different sectors of the IPV care network observed different manifestations of the underlying fear that pervaded the care environment. *“It felt like staff were concerned about their physical safety, so we [provided] services remotely.” (housing).*

“We saw a very dramatic reduction in our volume of patients willing to come into the hospital…they were afraid of the virus.” (healthcare)

### Community and system impacts

The pandemic highlighted and heightened pre-existing inequities and shortcomings in the IPV care infrastructure. Providers had to stretch already limited resources even more thinly to implement COVID-safe protocols and support and care for a larger survivor population. Challenges in supporting IPV survivors that had existed prior to the pandemic, such as finding adequate shelter, became only more difficult during the pandemic.

A. System-Wide Uncertainty and Inconsistency

Constantly changing public health guidelines forced institutions to update their policies frequently. The inconsistencies that followed made it difficult for survivors to access services. This was particularly evident within the legal system, *“We couldn’t keep up with who was supposed to do what in what court and how you applied…People just couldn’t navigate it…” (legal aid).*

B. Strained Systems and Strapped Resources

The pandemic worsened the already strained IPV infrastructure. Systems did not have the personnel, administrative or financial resources, or established emergency preparedness protocols to respond quickly and effectively to the sudden increase in need created by the pandemic, *“[COVID is] just magnifying the issues that were already in place: housing insecurity, food insecurity, access to medical care, racism…” (social work).*

III. Amplified Inequities

Communities of color and individuals experiencing economic insecurity were disproportionately affected by the pandemic. Participants involved in healthcare observed this: *“…racial inequality that has been exacerbated by COVID persists….My patients who live in certain communities … have a harder time engaging in care.” (healthcare)* We also heard this view from participants in other sectors, *“[COVID] is just magnifying the issues that were already in place: housing insecurity, food insecurity, access to medical care…COVID is disproportionately impacting communities of color…” (social work).*

IV. Loss of Community through Isolation

COVID-19 restrictions isolated survivors from their support systems (family, friends, community). *“This is an isolating hard time for everyone but especially for somebody coming from a relationship where they’ve been isolated with… very little community support.” (legal aid).*

### Individual impacts

Both survivors and providers experienced personal challenges throughout the pandemic, from worsening mental health, to reduced access to technology and childcare and school closings.

A. Heightened Consequences of Limited Technology Access

COVID-19 illuminated inequities in access to technology, which was essential for survivors to access care during the pandemic. Technology, in turn, became a necessity, *“…there is this expectation that like, ‘Oh, this is Zoom. Everyone knows how to do it. Everyone has that access to Wi-Fi at home,’ and that’s just not the case.” (legal aid).*

B. Complications of Childcare

Without childcare due to school and daycare closures, survivors with children faced the additional challenge of in-home schooling and full-time childcare, *“I’ve talked with a lot of survivors who get stuck in this pickle if they are displaced from their house, they’re trying to maintain their job while also taking care of their children while being remote…” (social work).*

III. Compounding Trauma

Providers noted that in addition to the physical and emotional trauma of abuse, the pandemic heightened other forms of trauma that many IPV survivors experience, including racial bias and poverty, *“Survivors are survivors of intimate partner violence, but they’re also survivors of intergenerational trauma, community trauma…state-sanctioned violence on our communities.” (social work).* Health care providers made similar observations, *“I think people who have experienced identities that have [been] oppressed have always been skeptical of any type of organized support, whether it’s medical, clinical, NGOs, because history has showed them that they’ve had to fight to be seen as equals, if not even just treated with the same care.” (health care).*

IV. Deterioration of Mental Health

Many individuals experienced a decline in mental health due to physical and social isolation, which was further exacerbated by limited access to mental health services during the pandemic, *“People have much less access to one-to-one private interactions with their therapists or their psychiatrists…They’re so isolated…Many of them are just sort of fraying around the edges generally.” (healthcare).*

E. Strain on Providers

Providers experienced some of the same stressors as survivors during the pandemic and felt “overwhelmed” by the work they were doing under these circumstances. *“We’re in COVID. Everyone is getting sick. Everyone is overworked and underpaid. Everyone is trying to get used to this new way of lifestyle that we weren’t used to before.” (social work).*

“I mean, it has been horrible. It’s just a gruesome time to be a health care provider…” (health care)

### Adaptations and innovations

To provide survivors with the care that they needed, providers had to be willing to modify traditional practices, adapt new treatment approaches (i.e. virtual interactions), be creative, and cooperate and coordinate with other providers involved in different sectors of survivors’ care.

A. Virtual Interactions

Virtual encounters allowed survivors to access care during the pandemic, where they would have otherwise missed out due to COVID-19 mitigation measures and the fear of contracting COVID-19. Virtual encounters also increased flexibility for providers and survivors in terms of scheduling and meeting. *“I would love that option to remain…it’s nice to have a little bit of flexibility, especially when you’re working with trauma survivors who may not get to court on time or may be facing all sorts of other barriers that makes it hard for them to get to the court on time.” (legal aid).*

Yet, providers reported drawbacks and shortcomings of virtual care too: difficulty building trust virtually, concerns over privacy during virtual encounters, and a loss of networking among colleagues that emerged naturally from casual in-person interactions pre-pandemic, *“It’s so much harder to make it an empowering and client-centered experience when it’s virtual.” (legal aid).*

“I think they don’t have the same opportunity for privacy that they get in the doctor’s office.” (health care)

B. Importance of Hybrid Care

The pandemic presented an opportunity for providers to move further towards a hybrid-care model (incorporating alternative care modalities, such as in-person, telephone, and video interactions), based on individual survivors’ needs, rather than a one-size-fits-all approach. Going forward, such a hybrid care model may help advance this more individualistic approach to care, *“I think having the choice is gonna be really important and being able to have clear criteria around which those choce are made, because …different patients have different needs.” (healthcare).*

III. Willingness to Modify Practices

The pandemic required providers to be flexible and innovative in engaging survivors, and to implement care modalities that were previously believed infeasible. In many cases, the benefits and increased access to care offered by these innovative techniques proved to outweigh the risks, highlighting the success of previously questioned strategies. *“We made it a point never to do telehealth before this…That’s quite different now.” (health care).*

“It used to be one of those things I [couldn’t] see how an advocate [could] work remotely, while they’ve been doing a darn good job of it. We have been using technology in a way that we didn’t really use it before.” (housing).

IV. Creativity

Providers had to alter their practices in fundamental ways to overcome the challenges of COVID-19, including utilizing virtual telehealth visits, Zoom-based court sessions, and temporary shelter in vacant hotel rooms.*“I think creativity goes a long way in this work.” (social work).*

E. Cooperation and Coordination

Despite the loss of in-person networking and interaction, providers across all sectors of care reported improving their communication and forming stronger relationships with other providers involved in IPV care during the pandemic, which they hoped would persist into the future, *“…we’ve created something called the Boston Partnership. It’s a collaborative with other domestic violence, sexual assault, and support entities within the city of Boston, so some of the hospitals, clinics, legal advocacy services are involved…it’s a way for all of us to connect and adapt to the changing system of COVID…” (healthcare).*

“…it has brought many of us closer together across organizations [through] our need to rely on each other.” (housing)

## Discussion

We conducted individual interviews with 18 Boston-area providers from several sectors—healthcare, social work, legal services, emergency shelters—with professional experience caring for IPV survivors. We sought to understand the challenges the COVID-19 pandemic presented in delivering IPV services and the ways in which providers adapted their practices to address these challenges. Providers reported that stay-at-home orders, social distancing measures, and economic insecurity, led to heightened violence and limited survivors’ ability to access care across all sectors, from housing support, to legal aid, to healthcare.

In addition to making it more difficult for survivors to access care, social distancing and COVID-19 safety measures also made it more difficult for organizations and providers to support survivors. Frequently changing, inconsistent public health guidelines added to the difficulties of providing care during the pandemic. Providers described the past year as “overwhelming,” “exhausting,” and “gruesome.”

At the same time, providers reported that open-mindness, creativity, and cooperation across different sectors of care were essential in enabling them to provide the best care possible to survivors throughout the pandemic. In particular, virtual interactions offered providers increased flexibility and allowed them to care for survivors whom they would not have been able to see in person. In some cases, providers even found virtual interactions preferable to in-person interactions, as they were more convenient and allowed survivors to bypass obstacles to care, such as transportation. Yet providers also emphasized that virtual care has drawbacks. Providers found it difficult to build trust and relationships virtually, and difficult to ascertain whether survivors were able to attend visits in a private and safe space, removed from their abusers. Going forward, providers hoped that virtual visits could be incorporated into a hybrid care model, one with the flexibility to tailor the type and medium of care (e.g. in-person, phone, video) to each survivor’s needs and circumstances. The authors have developed tip sheets for IPV healthcare providers during the COVID-19 pandemic that can be accessed at: https://www.brighamandwomens.org/assets/BWH/womens-health/connors-center/pdfs/covid-19-tic-booklet.pdf and https://www.brighamandwomens.org/assets/BWH/womens-health/connors-center/pdfs/intimate-partner-violence-virtual-inquiry.pdf.

In addition to the importance of virtual interactions, we identified several key themes: the importance of helping staff manage emotional strain and stress, the amplification of pre-existing economic and social inequities, and the heightened barriers faced by non-English speakers and immigrant survivors in navigating care.

The experiences and perspectives of providers included in this analysis may be used to inform decisions surrounding the provision of IPV services at a time when systems are emerging from strict pandemic restrictions and afforded the opportunity to reimagine standards of care. In this context, two themes, the importance of hybrid care and strain on providers, warrant further discussion. The element of flexibility is a primary advantage to hybrid IPV care, allowing providers to tailor service modalities to the individual survivor’s circumstances and maximizing the utilization and impact of those services. However, flexibility comes at costs, both economic and human. To offer in-person and virtual services effectively, programs would need to maintain in-person office spaces and equipment and provide reliable technology for remote engagement. Payers would also need to compensate providers in a way that incentivizes flexibility, rather than conventional service-delivery structures (e.g. only in-person services). Simultaneously, workforce fatigue and burnout must be addressed to ensure that providers are able to offer survivors the best possible care. Going forward, policy makers should factor in the impact of flexibility of care delivery modalities on provider burnout (and retention) when assessing organizational program resources and impact.

To our knowledge, this is the first qualitative study using long-form, semi-structured interviews with providers to examine how the COVID-19 pandemic impacted care for IPV survivors. Nonetheless, the themes we identified are consistent with findings of other work examining the impact of COVID-19 on IPV. Wood et al. report that among over 350 providers caring for IPV survivors during the pandemic, there was a 51% increase in video interactions with clients [17]. Wood et al. also report a decrease in available resources, an increase in demand for those resources, and an increase in job-related stress for providers during the pandemic [17]. In a qualitative analysis of housing providers, Nnawulezi and Hacskaylo found that helping staff navigate emotional turmoil and stress was a key concern among providers throughout the pandemic [18]. Finally, in a qualitative study of immigrant survivors in cities across the US, Sabri et al. report that immigrant survivors were less comfortable and less able to engage effectively with virtual resources offered in place of in-person resources than non-immigrant survivors were [10].

We acknowledge several limitations. First, the experiences and insights of providers working in Boston, which has a strong IPV infrastructure, may not be representative of those working in other, less well resoucred areas in the US. Further, participants in this study may not be representative of all IPV providers in the Boston area. In particular, given their willingness to discuss the subject of IPV care in depth, the participants whom we interviewed may be more willing to discuss creative and innovative approaches utilized than the average provider. Given our participants’ appearances in Google searches, responses to remote interview requests, and willingness to complete a telephone interview, they may also have greater access to, or ability to use, technology than the average provider. Second, we did not capture the perspectives of survivors themselves, who are key in IPV care, and whose experiences and insights are essential to the evolution and development of provider practices. Third, the breadth of our research question (how has COVID-19 impacted care for IPV survivors?) is reflected in our interviews and themes. Our comprehensive approach may come at the expense of a deeper understanding of individual facets of IPV care during the pandemic, such as hybrid care and its future applications. Finally, this work is qualitative and should be considered hypothesis-generating. We hope that our results will help guide future research and inform provider practices. Further investigation into the economic implications and cost-effectiveness of hybrid care may be particularly useful.

## Conclusions

This analysis has described significant tolls on survivors and providers during the pandemic. Our findings suggest that a hybrid care model that flexibly offers both in-person and telehealth care may help providers tailor their practices to each survivor’s experiences and needs to improve the quality of care they provide. Service changes made in light of the pandemic should consider the well-being of both survivors and providers. During the rapid expansion and alteration of IPV care during COVID-19, many providers personally filled gaps in services, which had serious consequences for workforce fatigue. Even after the pandemic, the creativity, cooperation, and coordination, which providers used to overcome the unprecedented challenges of COVID-19 will remain essential in providing survivors with care that is safe, equitable, and trauma-informed.

## Supplementary Information

**Additional file 1.** Thematic scheme with hypotheses and supporting quotations.

## Data Availability

The datasets used and analysed during the current study are available from the corresponding author on reasonable request.

## Abbreviations

IPV: Intimate partner violence
DV: Domestic violence
