# Supplementary material for: Provider perspectives on the provision of safe, equitable, trauma-informed care for intimate partner violence survivors during the COVID-19 pandemic: a qualitative study
Source: BMC Womens Health. 2021 Aug 27;21:315. doi: 10.1186/s12905-021-01460-9 (PMC8393774; doi:10.1186/s12905-021-01460-9)
Supplement: Supplementary file 1 — Additional file 1. Thematic scheme with hypotheses and supporting quotations. [file 12905_2021_1460_MOESM1_ESM.docx]

**Appendix 1.** Thematic scheme with hypotheses and supporting quotations.

| **Theme or sub-theme** | **Hypotheses** | **Supporting text from transcripts** |
| --- | --- | --- |
| **I. Pandemic Threat** |  |  |
| Exacerbated External Stressors | The increased stress of economic and housing insecurity, in addition to the stress of the pandemic and increased isolation, produced circumstances that increased the likelihood of violence from abusers. | “Housing security is incredibly important. Boston has a extreme crisis of affordable housing. That is one of the reasons why there is an uptick—not just during the pandemic, but in the past five years, if not longer—of experiencing sexual violence and intimate partner violence because new immigrants—people who are not making a living wage. Not talking about minimum wage. I’m talking about a living wage to survive in this city. Recent grads especially. Those three areas are particularly vulnerable and often find themselves in sub-standard or unsafe housing situations because it’s the only financial option. I think affordable housing and accessible safety net programs that not only address the symptoms, but the cause, will be what is a huge way to both prevent and protect situations of intimate partner violence.” (27)  “I would say, compared to an economic crisis, I think it’s very similar in that people are not able to make—all the folks that were basically on the cusp of survival now have fallen off that, and this is their breaking point and increased homelessness, et cetera, all of which we know increases stress within a relationship and then also increases vulnerability to intimate partner violence.” (55)  “I think for the perpetrators— this big economic stress is a big stress on perpetrators, right? They lose their job. They lose their identity, and they’re home with children in a small space, and I think you could see the writing on the wall that it’s not gonna go well.” (58)  “This is a pandemic, so we don’t go into people’s homes, but parents will bring kids down to the van, and we have it set up like an exam room and then just understanding that it sucks to be poor, and it sucks more to be poor during COVID. [Laughter] Nobody has a cushion to fall back on if your baseline is that you’re living paycheck to paycheck, and there’s no paycheck, that it’s incredibly stressful for families.” (58) |
| Restricted Access to Healthcare | The emergency department (ED) was not easily accessible to individuals experiencing violence because of the COVID-related restrictions on transportation and capacity.    Fear of contracting COVID prevented some individuals experiencing violence from accessing health care.    Support systems for survivors who sought medical care were severely limited, as companions were not allowed in the ED and visitors were not allowed in the hospital. However, visitor restrictions also provided more opportunity for doctors to screen for violence without a possible abuser in the room. | “There’s no way to go to the doctor and speak with your PCP and tell them that . . . I’m in an abusive relationship. No one is allowed to go to the hospital unless it’s a major issue.” (51)  “Some of that is they don’t want to [come into the hospital] because they’re afraid of the virus. Some of it is because we were under pressure to decrease our in-person footprint just for the safety of the people that did come. Sometimes, it’s something as simple as transportation. They have no way to get in to see us.” (58)  “We saw a very dramatic reduction in our volume of patients willing to come into the hospital. People didn’t wanna bring their kids in because they were afraid of the virus.” (58)  “On one hand, it’s actually been great for screening for violence because you don’t have to kick anybody out of the room. You don’t have to get creative. . . . You’re able to talk to the patient alone without any extra logistical or interpersonal funkiness.” (55) |
| *Fewer Interactions with Mandatory Reporters* | *Due to physical isolation during the pandemic, survivors were seeing fewer mandatory reporters, such as doctors or teachers. This resulted in fewer reports of IPV through these channels.* | *“We are very worried about that, about people not connecting with services, and we do know that DCF has received many fewer reports of child maltreatment during the pandemic because a lot of the mandated reporters are not seeing the kids.” (58)* |
| Fewer Opportunities to Leave Abusive Environment | During the lockdown, many survivors were forced to remain in close proximity to their abuser, which decreased their ability to escape or access resources.    The fear of COVID and public health guidance to combat the pandemic gave abusers leverage for increasing their control over survivors. And as a result of social isolation, abusers were able to cut off their partners from social support systems to an even greater degree.    The absence of social or emotional outlets led to a breaking point several weeks into the pandemic. No longer able to cope with their situations, survivors turned to hotlines and shelters. | “The next issue was making sure that folks were secure and stable. That came in a lot of different situations and variables because when we had a stay-at-home order in April, what that does, is you’re at home with your abuser. When you’re asked to stay at home with your abuser in a stressful situation, issues of violence go up for obvious reasons that I don’t need to spell out to you ’cause you do the work too.” (17)  “Our impression is that it’s worse, that people are not getting services and that they are trapped with their perpetrators in uncomfortable situations where people are stuck inside.” (58)  “This is an isolating hard time for everyone but especially for somebody coming from a relationship where they’ve been isolated with very limited access to resources or very little community support if any because their abuser did a really good job of cutting them off from their community support or took them from a place that they know.” (53)  “We have heard from advocates who we have spoken with. In general, through faith communities, we know that people who are experiencing abuse now with the—it was really bad when there were stay-at-home orders in most states. Even now, when there aren't the same stay-at-home orders, people are still staying at home a lot more with social distancing, and things aren't open the way they used to be, and especially now that it's getting colder and people aren't out as much, there aren't as many opportunities for people to—if you're working at home and you're working under the same roof as the person abusing you, you're not getting any breaks.” (31)  “Once people figured out the flow of things and also once the proximity started to escalate some of the danger, the phone was ringing... What we were hearing in a lot of the calls that were coming through, the new calls for service was a lack of ambivalence. People were calling and saying, I need to get out now. I need to do this now. I need to do this. I need to figure something out now. As we reflected on it, we realized that people who often have this valve, whether it's a family member or friend or an activity that gives them space from the abuser or a place to go when things you know, things get intense and dangerous. That was no longer available. They weren't able to go sleep at their mother's house or go be with a friend or take the kids and go visit their sister or brother. They were stuck. That lack of an escape valve and lack of options really, for a lot of people, crystallized and focused them on doing something right away.” (43) |
| Fear of COVID-19 Infection | Providers feared for their own safety from COVID exposure, which promoted a speedy transition to remote work and virtual encounters.    Survivors were fearful of contracting COVID. That fear often decreased their willingness to access resources. | “It felt like staff were concerned about their physical safety, so we’re able to fund and house eight families and provide services remotely.” (45)  “At the beginning, it was a little bit harder to set up that routine ‘cause I was so nervous about COVID in my own family and my community around me.” (3)  “We have a hard time with staff willing to come on site. Then, people were just willing to quit or lose hours and just not wanting to come to work.” (45)  “I think the people who were in the shelter didn’t feel safe because they didn’t feel like they could protect themselves from the virus.” (43) |
| **II. Community and System Impacts** |  |  |
| System-Wide Uncertainty and Inconsistency | The constantly changing public health guidance and policies surrounding COVID meant that providers and systems were in a similar state of flux throughout the pandemic. In-person services, like courts and shelters, were especially affected.  The constantly changing COVID policies made it hard for systems to adapt or communicate their new policies effectively. | “...to have a system be responsive in the middle of a pandemic is almost an impossible job, and so every time something changed, the courts’ instructions would change.” (25)  “Sometimes the instructions would be different from one court to another. You had attorneys that were scurrying to try and figure out how do I file this, how do I file that? In March, it was one answer. In June and July, there was another answer. That’s to the court’s credit because they were responding to a change in the virus. By the same token, if you have to figure out the rules every time you’re gonna file something, it slows everybody down. It’s just much more complicated, and the courts can’t handle the same volume that they could pre-pandemic.” (25)  “I think the courts would be, if they could have a uniform response to things, I think that would make things a lot easier.” (52)  “As a lawyer, it was so confusing what the courts wanted to do. There’s all these—there’s probate and family courts, district courts, and the Boston Municipal Courts are three different systems. We couldn’t keep up with who was supposed to do what in what court and how you applied. In some courts, it was email, and some still wanted it in person but you couldn’t go in, but you handed the paperwork to a guard through the door. People just couldn’t navigate it and having the lawyers, to some degree—we would generally know who to email to get this—with this issue.” (48) |
| Strained Systems and Strapped Resources | Established systems did not have the bandwidth, surplus of resources, or established emergency preparedness protocols to quickly and effectively respond to a sudden and large increase in need during the pandemic.  Some providers feel that systems were already failing to provide adequate resources before the pandemic, and the pandemic only worsened an already bad situation.  Organizations that had a reserve fund or endowment were better able to continue providing services during the pandemic because they had the financial flexibility to do so. | “What are the lessons? I think we should have more money available because we've incurred lots of expenses that I had not dreamed we would have had when I made up the budget for the year.” (38)  “I go back to thinking about domestic violence being an issue that happened before COVID and not having enough resources and support on a systemic level, for housing, for food security, for all of our basic needs. When something like this happens, I think the response should always be from a larger scale level from a macro level, for the government, for the city of Boston, for the mayor, to always keep an account that what survivors need, and what communities just need in general, to create safer communities. To me, that's always going to go back to providing what families need, what communities need to be safe: food, housing, education, Medicare, all of that.” (Participant 3)    “For me, it's always gonna go back to COVID is showing us how–– it’s just magnifying the issues that were already in place: housing insecurity, food insecurity, access to medical care, racism, all of that is just magnifying all of that. COVID is disproportionately impacting communities of color because of racism, because of capitalism, because we live in a society that puts profit over human need... They say these systems are supposed to help us and help the community, but instead, what we experience is, for example, like different housing programs or like DHCD, while they are here to help house families, it is so difficult and my clients struggle to get into shelter. They feel that there are a lot of barriers to get into shelter because they're being denied.” (3)  “Yeah, I’d say as a nonprofit, I think it becomes increasingly important to have a reserve fund or an endowment available for emergencies like this. We were lucky because we’re such a large nonprofit, and we do have a board advised fund where should we need it, we have about a million and a half to fall back on. It really allowed for stability of funding, stability of access, stability of services, and we could make more survivor-led decisions because of that. Then to me, it increases the importance of having relationships with policymakers both city, state, and federally even if you are a local organization because, without it, we wouldn’t have been able to access emergency funding. There’s a really good bank so that we can get access to that PPE really quickly.” (17) |
| Amplified Inequities | Those who were already experiencing the greatest oppression and insecurity were the hardest hit by the pandemic. In particular, communities of color and individuals living in poverty experienced extreme difficulty dealing with the challenges of the pandemic. | “[COVID] is just magnifying the issues that were already in place: housing insecurity, food insecurity, access to medical care, racism, all of that is just [magnified]. COVID is disproportionately impacting communities of color because of racism, because of capitalism, because we live in a society that puts profit over human need. . . . I’m doing this really, really hard work with survivors, where I’m coming against systems that are consistently pushing back.” (3)  “There is no question that COVID has heightened a lot of inequalities and by heightening those inequalities, that means that people who are experiencing IPV--I mean the more resources you have at your fingertips, the easier it is to get the resources that you need to deal with whatever it is.” (31)  “With the Zoom school world for low income, mostly immigrant populations, especially folks that have been extremely isolated in their relationship, and now that they’re out of their relationship or exiting their relationship don’t necessarily have access to resources.” (53)  “From an inequities perspective, I think I call the pandemic the great reveal. . . . Everything we knew was there, it just yanked the blanket off the top of it.” (43)  “In some ways, COVID has heightened the inequalities and made it harder for people who do experience multiple levels of trauma not to be able to get to the resources that they need, but then at the same time, if you’re being controlled by somebody, you’re being controlled by somebody.” (31)  “While we’re broadening access in some ways, we’re not broadening access equitably, and the racial inequity that has been just exacerbated by COVID persists. I see this same racial inequity repeated as I look at my patient population. My patients who live in certain communities I feel like have a harder time engaging in care.” (59) |
| *Language Barriers to Accessing Resources* | *Those who don’t speak English were less able to access resources that were available during the pandemic.* | *“Then, for clients for whom they could access emergency RAFT rent relief in the Greater Boston area--if they didn’t speak English as a first language, if they were unfamiliar with how to apply for it, if they didn’t know to ask for it and we didn’t know to offer it, they were inherently less likely to be able to apply for, be accepted to, and have access to emergency rent relief funding.” (49)*  *“It’s always been a disadvantage, I think, when you have an interpreter to present your case and make the judge kinda--you want the judge to feel for the person and I think it’s hard to do that when you can only speak 10 words at a time and then pause and wait for the interpreter. It’s hard to convey emotion and stuff through an interpreter.” (48)* |
| Loss of Community through Isolation | Through the experience of social and physical isolation, survivors were deprived of community even after leaving a violent situation. COVID restrictions barred them from being able to gather with friends, social contacts, or neighbors, essentially cutting survivors off from their support systems. | “Many of them live in shelters or congregate living environments, and they’re afraid so they stay in their rooms, which is just terrible for their babies.” (58)    “Yes. That's the biggest part with people, that isolation. That is one of the things that we are seeing, depression. Depression just has been huge.” (5)  “Well, I think one of the biggest things, and it’s conversations that I’ve been having across different IPV agencies or DV agencies about the fact that people are way more isolated right now than they ever have been.” (52)  “I think if there's—having ways for survivors to connect more because it is an extremely—it's an extremely isolating time for anyone, but especially if you're in a situation of IPV.” (50)  “In addition to that fact—my patients who are isolated to begin with are even more isolated, even more lonely and in that setting, because psychiatry and psychotherapy have decided that they are not essential. They are there seeing people in person every six weeks where I work. People have much less access to one-to-one private interactions with their therapists or their psychiatrists. What I am seeing in my already isolated—you know, they’re already isolated. For many of them, they don’t mind that, ’cause they don’t really care about social—it’s complicated, but anyway. They’re so isolated, and they’re even more isolated now. Many of them are just sort of fraying around the edges generally.” (54)  “I would say one of the biggest crimes society commits against survivors is gaslighting. It is questioning your sanity and your experience because it’s easier than making hard changes and calling out violence when it is seen. That’s not different because of the COVID-19 pandemic, but you may have fewer people in your life who are part of your social circle or safety net. Isolation is one of the hardest parts of experiencing assault, sexual violence, intimate partner violence.” (49) |
| **III. Individual Impact** | | |
| Heightened Consequences of Limited Technology Access | For both providers and survivors, access to technology was essential to continue working and connect with resources, respectively, during the pandemic. Pre-existing inequalities in access to technology and Internet left individuals with limited access cut out of the care system.  Many providers have begun to think of technology as a basic utility following their experience during the pandemic. | “One challenge is there is this expectation that like, ‘Oh, this is Zoom. Everyone knows how to do it. Everyone has that access to Wi-Fi at home,’ and that’s just not the case.” (53)  “Then again, a lot of lower-income people don’t necessarily have a good laptop, or their kids are using the laptop for school.” (48)    “We’re supposed to be getting new—all staff had laptops before this, and we had smartphones for staff, which was something that was planned was how to execute it, but yeah, trying to still understand what the responsibility is. I was thinking of the worst for survivors and because we work with such a large, particularly immigrant community where one family shares a phone.” (45)  “Then I think the other piece of it is that it was so clear working with the families that we were working with that you know, there were so many families. They have four kids in four different school levels, and they have one room and one computer.” (43)  “It's like how are these four children supposed to get their education when you have one computer? You don't have a data plan either 'cause you can't afford it, and so helping people access the Internet, the community Internet who's made available, but still the differences that are just so clear and to somehow establish the public school to be delivered in this way when people do not have equitable access to the means of delivering it, I mean that's just unacceptable.” (43) |
| Complications of Childcare | For survivors with children, the pandemic posed added stressors and challenges as they had to adapt to in-home schooling and limited access to childcare.  Survivors with children faced the additional barrier of acquiring childcare when trying to access in-person resources, such as the ED or court system. | “I’ve talked with a lot of survivors who get stuck in this pickle of they are displaced from their house, they’re trying to maintain their job while also taking care of their children while being remote, while trying to be in a safe place.” (50)  “People who are parenting have been squeezed in ways that--I can say first-hand as a parent, but beyond that, from when I see my patients who are parents--in just untenable ways.” (59)  “We noticed it, as providers, in a place where some of our docs with the young kids, they had the resources to create a pod for their kids or their kids go to private school where they’re going in person, and none of the kids in the public schools in Boston are going to school in person and so that just increases the stress.” (58)  “I don’t know what percentage of people obtaining restraining orders have children, but I would say at least 60 percent, if not more. My guess is the majority are single parents, and it’s unreasonable to ask single parents, right now, to do anything involving going places, especially single parents in poverty.” (53)  “Moms who are experiencing IPV that need to come in to health care--they have a medical problem that’s potentially severe. They now have to totally figure out childcare, ‘cause in the past, they could bring their kids back with them, right? . . . I do know, with the increase in access to telemedicine, issues around transportation and childcare are less for those that are already plugged into our system, but some of our most vulnerable victims of IPV are not already tied into the system in those ways that allow an easy telemedicine visit. For those individuals, then there’s this added layer of like, ‘Well, what am I gonna do with my kid if I’m not allowed to bring them back?’ So maybe they avoid it altogether.” (55) |
| Compounding Trauma | Survivors of IPV are likely experiencing other forms of trauma, such as the trauma of oppression or poverty, in addition to the physical and emotional trauma of abuse. All forms of trauma were heightened by the pandemic. | “I think this year. . . has shown everything about the country that we’re living in. We’re talking about the attacks on immigrant people. We’re talking about the attacks of police brutality on people of color, on Black and Brown people. Survivors are survivors of intimate partner violence, but they’re also survivors of intergenerational trauma, community trauma, and where I’m not just working with them on ‘Oh, what’s going on with domestic violence.’ We’re talking about community violence. We’re talking about state-sanctioned violence on our communities. When we put all of that together, my response is gonna be we need a system that cares and prioritizes human needs, and not just some human beings, but all of us starting with the most marginalized.” (3)  “I think we’re talking about what oppressed communities have already been experiencing. . . . We have been experiencing the pandemic or the epidemic of racism, oppression, xenophobia, sexism, all of these things, has always been a thing, right? Then when you add COVID to it, it just exacerbates that.” (3)  “I had a number of clients who are Black over the course of the summer. Everything we would talk about would be additionally made so much more painful by the repeated re-traumatization, and incredibly forefront in the news and the media. Polarized opinions on if Black people deserve human rights.” (49)  “I think people who have experienced identities that have oppressed have always been skeptical of any type of organized support, whether it’s medical, clinical, NGOs, because history has showed them that they’ve had to fight to be seen as equals, if not even just treated with the same care.” (49) |
| *COVID as Trauma* | *The experience of living through a pandemic was traumatic in and of itself for both providers and survivors.* | *“When I think about survivors of domestic violence, and what they have already experienced when you add COVID and when you already add the trauma that they have already been living through, it just exacerbates depression, anxiety, and other mental health concerns as well.” (3)*  *“Yeah, it’s definitely not easy. When we’re advocates or providers of color, and we belong to different groups as well. Social workers and advocates, many of us go into the field because we want to help because we have similar lived experiences. We also have trauma that we have worked through or continue to work through. I think it has impacted all of us.” (3)*  *“I think the last year has been really hard and overwhelming and exhausting.” (45)*  *“I mean, it has been horrible. It’s just a gruesome time to be a health care provider.” (58)* |
| Deterioration of Mental Health | Mental health services were in greater demand during the pandemic, exacerbating a pre-existing shortage of mental health care providers.  The forced physical and social isolation many experienced during the pandemic could cause a deterioration of mental health, especially among individuals who already struggled with mental health. | “My people ... sometimes they just see their psychiatrist every three months for 15 minutes to check in about their medication, but they come in and say, ‘I haven’t heard from Dr. So-and-So.’....the quality of connection they get from having a PCP or a psychiatrist or therapist who actually cares about them at all, it’s just really different from the staff at the group home who in some cases are amazing. Some cases are just punching a timeclock.” (31)  “In addition to that fact—my patients who are isolated to begin with are even more isolated, even more lonely and in that setting, because psychiatry and psychotherapy have decided that they are not essential. They are there seeing people in person every six weeks where I work. People have much less access to one-to-one private interactions with their therapists or their psychiatrists...They’re so isolated, and they’re even more isolated now. Many of them are just sort of fraying around the edges generally. Their mental health status is declining in addition to their having much less opportunity to come forward with difficult things that are happening to them—” (54)  “This is an isolating hard time for everyone but especially for somebody coming from a relationship where they’ve been isolated with very limited access to resources or very little community support if any because their abuser did a really good job of cutting them off from their community support or took them from a place that they know.” (Participant 53)  “That's the biggest part with people, that isolation. That is one of the things that we are seeing, depression. Depression just has been huge. Also, what we try to do as staff members, we check on them constantly, especially if we know someone already has suffered from depression. We check on them.” (51) |
| Strain on Providers | Providers experienced many of the same stressors as survivors during the pandemic and often found it difficult to maintain their own well-being while trying to care for survivors.  Many providers felt “overwhelmed” by the work they were doing during the pandemic and needed to implement changes in their own lives to cope with that feeling. | “Yeah, it's definitely not easy. When we're advocates or providers of color and we belong to different groups as well. Social workers and advocates, many of us go into the field, because we want to help because we have similar lived experiences. We also have trauma that we have worked through or continue to work through. I think it has impacted all of us.” (3)  “...it can be overwhelming. I think that’s something to highlight because I’ve heard that across. Not to take away from the survivors we’re working with, but I think highlighting the stress bubbles that I think the people that are doing the work that they’re facing also is important to acknowledge.” (52)  “In that way, those things like I'm happy that I moved, I think it's overall a better feeling, set up different office space. I continue to schedule my days in a certain way, so that I don't feel completely overwhelmed. I think, at the beginning, I felt very overwhelmed and just learning through the process, how to better schedule my days because the need was so much higher, and the intensity of the work.” (3)  “We’re in COVID. Everyone is getting sick. Everyone is overworked and underpaid. Everyone is trying to get used to this new way of lifestyle that we weren’t used to before.” (3)    “I think some of the stuff that we learned is not only impacting how we're doing that work with providers, but also with ourselves in terms of addressing overwhelm and burnout and thinking about those strategies.” (43) |
| **IV. Adaptations and Innovations** | | |
| Virtual Interactions | All providers pivoted to some version of hybrid or entirely remote work with at least a portion of their survivor interactions being virtual.  Virtual encounters were highly efficient in that, on average, more survivors were able to access services remotely.  Virtual-only encounters could be useful for sharing information with survivors but weren’t as effective at ensuring understanding of that information. | “We have been using technology in a way that we didn't really use it before. We've been doing all our staff meetings using Zoom and we've all become very accustomed to speaking on Zoom.” (43)  “There’s absolutely upsides. Our adolescent medicine people are really busy with telemedicine visits. The teenagers really seem to like them, and our no-show rates are much lower for the teenagers on the telemed platform than they are for in person. I do. I think the world has changed and that a lot—not a lot, but probably, I would guess somewhere around 25 percent of our visits moving forward will end up being over telemedicine.” (58)  “When you have a phone conversation without having seen the person first, it’s just a disadvantage, and it takes away—I think when people connect with each other, they have a visual. If they never have the visual and they’re only talking by phone, then it depends on the nature of advice that they’re seeking... If somebody’s talking about their own personal story and you need to help them write an affidavit for court, for example, then I think that it’s not gonna be as effective over the phone unless you had Zoomed with them first and they can picture you.” (25)  “I think it’s both. I think the access to technology—people are able to have a Zoom call in a space where they can actually focus and be present for the meeting is really rare. Those people are doing three other things, and so if you’re trying to talk about your history of trauma—and the other things just being, in general, the relationships are so different and being able to provide support. The depth of conversation that you get via Zoom is much less.” (45)  “It’s so much harder to make it an empowering and client-centered experience when it’s virtual and also just understanding the neurobiology of trauma and how it impacts our clients’ ability to retain information, remember timelines, keep track of things, how folks are navigating that with remote learning. I have had times where I’ve felt I’ve been very clear with a client on the phone, and I’m taking clear notes. Then, just I think because of trauma brain, my client has had a harder time absorbing it, and it’s hard to tell that on the phone and not able to see a person’s expression and see their comprehension and their comfort level to conversation. I think that that is an important thing, first, for folks working with survivors or really, any sort of trauma to figure out how to better navigate.” (53) |
| *Flexibility of Virtual Encounters and Remote Work* | *Having the option of virtual encounters added a layer of flexibility for providers and survivors that, in some cases, increased access to resources. Survivors who would otherwise miss out on encounters entirely (i.e. due to lack of transportation or childcare) were able to at least have a virtual encounter.* | *“They were appreciative of being able to fit it into their schedule as opposed to giving up a whole day for a training. We were able to reach people who are further out who wouldn't have been able to drive to a training in any area, but they were able to connect online. There were definitely some advantages.” (31)*    *“I would love that option to remain. I think that there is some nice—it’s nice to have a little bit of flexibility, especially when you’re working with trauma survivors who may not get to court on time or may be facing all sorts of other barriers that makes it hard for them to get to the court on time.” (53)* |
| *Difficulty Building Relationships Virtually* | *Providers found it difficult to establish trust and build relationships with survivors through virtual encounters alone.*    *Lawyers find it more difficult to assess how a survivor may appear to a judge in court when only virtual encounters are possible.* | *“I think that there's something, like I said, to being one-on-one and sitting next to each other crying—not that crying is good, but that you're able to be really feel seen in this setting and the privacy that people just do not have access to in a lot of cases, you know the dignity and it's their special place and there's privacy that our office has been able to offer is not something that can be replicated on Zoom.” (43)*  *“Really with DV survivors, you really do depend on building up a trusting relationship with people. It’s hard to do that without in-person—it’s just harder without in-person contact.” (48)*  *“I found that at the beginning, it was really hard for me personally, and it was really hard for my clients as well especially because we're so used to seeing each other face to face. Being face to face with a client brings us intimacy that I sometimes didn't feel that I felt over the phone. Personally, I felt really sad that I couldn't be there with them especially also because of COVID and my clients who were calling me while they were sick. For both of us, for me and my client, it was really hard to not be there in person, and we processed that about how is it for you that I can’t see you in person and that we have to see each other through a screen? At the beginning, it was very difficult.” (3)*  *“I had a client who was extremely emotional and would start screaming when she would talk about the abuser. We were preparing for a hearing in probate court by Zoom, and I said to her—because I had been able to see her on Zoom myself in terms of working with her, I was able to say, ‘You can cry, but don’t—you have to cry with some reserve. You can’t just cry with abandon ’cause the judge is gonna not understand where it’s coming from.’ I think you have to be able—part of what a judge does is he assesses or she assesses the credibility, and so to have a person appear in Zoom is very important. I think that the attorney needs to be able to see that and have enough experience with the client visually so that they know what to anticipate and what instructions to give.” (25)* |
| *Privacy Concerns* | *When accessing virtual resources from home, survivors oftentimes do not feel safe or lack the privacy necessary for them to be fully open and honest.*    *Providers worry about taking privacy and safety for granted during in-person visits. Over video and phone, providers can’t know who else is in the room and need to establish safety and privacy at the beginning of every visit.* | *“Also with the teenagers—and when you talk to one our adolescent medicine docs you’ll probably hear this directly. They’re less inclined to do a video visit. A lot of them just want to be on the phone and when we’ve tried to understand that, some of it, we think, is that a lot of our families live in very crowded conditions, and there’s some wish for privacy or maybe embarrassment about their living conditions.” (58)*  *“We ask people very explicitly anytime we do an intake or if there’s any concern if they’re in a safe place to talk. If they say no, we end the conversation immediately. We give them tools to be able to advocate for themselves, such as a codeword or a password that they need to say before they hang up the phone if there’s something that’s unsafe happening. For the most part, if there’s a situation where a client is in an unsafe place, we would see them in person.” (49)*  *“On the other hand, if they’re sitting at home and they don’t know if their partner is overhearing or even if their kids are overhearing or even a random—or whoever, their parents or their group home housemates, I think they don’t have the same opportunity for privacy that they get in the doctor’s office.” (54)*    *“With the advent of telehealth, we had to create guidelines around screening for IPV and stress the importance of it. We typically tell all providers at the hospital that if you're screening to do it alone. Make sure that the survivor is alone.” (50)*    *“I think there really isn’t an expectation of privacy for people who are living in an actively violent relationship or an actively abusive relationship.” (17)* |
| *Loss of Networking* | *During the pandemic, providers no longer had in-person conversations and casual interactions that could lead to unexpected connections and networking.* | *“Nobody knows what other people are up to, and it changes day to day. I’m like, ‘I think you can call these folks, and I think that they’ll provide some services,’ or I don’t know if you work with them, but I was like, ‘Oh, make a referral’…It’s harder to network with somebody on Zoom. I remember I used to go to meetings or sit next to somebody and be like, ‘Oh, yeah. I actually have a client who speaks Russian and needs DV legal services. I’m so glad I sat down next to you,’ but that doesn’t happen.” (53)*    *“Before the pandemic, we would have the meetings once a month and go through the business—the cases we would talk about. It would be just about the cases, but then naturally, at the end of it, you kinda chat and just talk to the different providers and maybe different things that weren’t on the meeting business but oh, I have this case…On Zoom, that’s the chit-chat that I think is really important for getting to know community providers. It’s a lot less natural on Zoom.” (48)* |
| Importance of Hybrid Care | The pandemic provided an opportunity to move towards a care model based on individual survivors’ needs and away from a one-size-fits-all approach. The emphasis was placed on meeting survivors where they were.  The uncertainty and constant change of the pandemic forced most work to be centralized through an advocate rather than being shuffled among various organizations.    Innovations that arose during the pandemic, such as virtual encounters and remote work, may provide necessary options and flexibility for survivors and providers in the future. Some survivors may prefer virtual visits while others might not. Having both as options may increase utilization. | “There’s so many different things that go into this. Complex trauma can be a part of it. I think just really meeting the person where they’re at, but always starting with that emotional and physical safety planning, but support is where it really starts.” (52)  “I think having the choice is gonna be really important and being able to have clear criteria around which those choices are made, because even within psychiatry and even within my patient panel, different patients have different needs. Some clinically really need a more in-person modality, and others really strongly prefer virtual and are doing fine clinically, and still others are actually doing better with virtual care.” (59)  “I will say the service providers are really doing everything they can to make sure that victims are getting what they need. Whether it’s something just as getting them connected to food stamps or transitional housing or things like that, or getting them the services they need for getting a restraining order or support.” (31)  “I would love to go back in person because I love especially when I run groups and everything like that, I think it’s important to be face to face, particularly with some clients that need extra support around, emotional support and psychological support. Also there are clients that for one reason or another, they cannot make it face to face. It’s more convenient for them to have a phone call or a virtual session. I think it’s possible to do a face to face and also be flexible for survivors who can’t come face to face.” (3)  “Yeah, you don’t have to spend all day in court. It can be quicker in a way. For some clients, it’s not so terrible. I think what has to be done is that you have to provide options. I don’t think that Zoom is a bad thing necessarily.” (25) |
| *Emphases on Survival and Emotional Support* | *Some providers noticed a change in the nature of conversations with survivors during the pandemic. Survivors were concerned about securing basic necessities (i.e. food, housing) and were reaching a breaking point in terms of dealing with the added stress of the pandemic.* | *“Now, so much of the work has been done around basic needs, helping people apply for cash assistance and just general emotional support, talking with people about--now they’re getting COVID tests for their family and helping people figure out funerals and bringing them resources for family members who have died from COVID because our work is to support people on all major things. So much of that has been helping people with remote schooling and their children and just talking about things like that as well so the type--how we’re spending our time with people has really changed for people who are really currently in abusive situations.” (45)*  *“Then after that, when people were home for a while, and it was just this monotony of unknown, we provided a lot of the emotional support, which we hadn’t anticipated to that degree. Frequently, we’ll be on the phone with survivors for anywhere from 15 minutes to an hour. Those calls were stretching out to one, two, and three hours on occasion.” (17)*  *“Really, it’s providing that emotional support that they don’t have access to in other places.” (17)* |
| *A Refocus on Basic Needs* | *Many survivors faced food and housing crises during the pandemic. Providers thought that work in these areas should be prioritized to ensure that survivors can meet basic needs.* | *“Now, so much of the work has been done around basic needs, helping people apply for cash assistance and just general emotional support, talking with people about--now they’re getting COVID tests for their family and helping people figure out funerals and bringing them resources for family members who have died from COVID because our work is to support people on all major things. So much of that has been helping people with remote schooling and their children and just talking about things like that as well so the type--how we’re spending our time with people has really changed for people who are really currently in abusive situations.” (45)*  *“The other thing that we’re hearing is that they are also getting calls--when they do get calls, sometimes it’s for really basic stuff. It has nothing to do with the abuse. . . . No, it’s more, ‘We have no money. We both lost our jobs.’ They can’t even focus on the abuse right now. They’re just looking on to survive day-to-day.” (31)* |
| Willingness to Modify Practices | The COVID-19 pandemic and its restrictions pushed providers and organizations to implement policies and ideas that were previously considered impractical or unfeasible. | “We made it a point never to do telehealth before this…That’s quite different now.” (49)    “It used to be one of those things I don't see how an advocate can work remotely. [Well] they've been doing a darn good job of it. We have been using technology in a way that we didn't really use it before.” (43)    “I think the courts have, for the most part, gotten pretty good on some of the technology stuff. They just had to switch to doing hearings first by phone and then by Zoom. It actually is kinda nice for a lot of reasons...” (48) |
| Creativity | Providers had to fundamentally alter their practices to overcome the challenges of COVID, which required creativity and adaptability. In particular, providers were creative in the ways that they brought services to survivors, including through virtual visits, mobile clinics, and Zoom court proceedings. | “Along with a number of other programs across the state, we depopulated our shelter a couple weeks into the pandemic by putting families up in a hotel that was designed for extended stay, so with a kitchen, that kind of thing, and found a hotel that no longer had any other clients.” (43)    “We developed a mobile vaccine program and we brought vaccines to kids at home. We’ve given like 3,000 vaccines since April to kids in the city of Boston by bringing—we partnered with an ambulance company, and a pediatrician and a pediatric nurse go out every day. We vaccinate kids who, for one reason or another, are not gonna come to the hospital.” (58)  “The courts have adapted, sort of, with Zoom—some of them. Some of them are still really resistant to Zoom hearings and really want in-person stuff, which they say—I think the courts are saying is in part because some of the buildings still aren’t Wi-Fi-enabled, apparently, in some of the courthouses, they say.” (48)  “I think creativity goes a long way in this work.” (50) |
| Cooperation and Coordination | The challenges of operating during COVID forced an increase in cooperation amongst providers.  Many providers agree that relationships built during the pandemic can persist beyond.  Communication among providers within and across care sectors improved during the pandemic, as they were forced to overcome the limitations of not interacting with each other in person. | “I think the helpful thing is being really submerged within the health center makes a huge difference. Even when we’re not on-site, we still have that connection to the [redacted for de-identification] to providers that we’ve formed relationships with which has really been helpful to keep the referrals going.” (Participant 52)  “I think the last year has been really hard and overwhelming and exhausting. Some of us were joking that it has brought many of us closer together across organizations like our need to rely on each other. I feel like I’m working a lot longer hours. I’m working a lot more. I thought I was busy before, but also it’s like because it takes time to reach out and how we’re still trying to do that, ask for support. I’m like, “’What’re you doing about this?’ In some ways, I feel like it’s created deeper connections, but it’s—if you’ve been intentional about doing so. I think it just highlights the importance of relationships of all kinds.” (45)  “The increased collaboration that I referred to earlier that I think happened whenever a crisis occurs, you just kind of—you forget your politics, you forget all these other sort of quarrels with other organizations, and you’re just like, ‘We have this common goal.’ I bet relationships will persist. I bet that those foundations and the ways that they were able to kind of work together will continue to bear fruit in a positive way, so that’s one thing that I’m hopeful is a silver lining.” (55)  “Yeah, I think the largest is we’ve created something called the Boston Partnership. It’s a collaborative with other domestic violence, sexual assault, and support entities within the city of Boston, so some of the hospitals, clinics, legal advocacy services are involved. Essentially what it is, it’s a way for all of us to connect and adapt to the changing system of COVID and, most specifically, in access to housing.” (17)  “We have partnered with more agencies than before, but not necessarily across different—I can't think of the word. We're not partnering with the courts, we're not partnering with law enforcement or anything like that. We're partnering more with different organizations. That's always our mantra is that this idea of a coordinated community response is huge and more important now than ever because you just don't know at what point where someone's gonna come into the system or something's gonna come to light.” (31) |
